# Gut Health-Promoting Benefits of a Dietary Supplement of Vitamins with Inulin and Acacia Fibers in Rats

**DOI:** 10.3390/nu12082196

**Published:** 2020-07-23

**Authors:** Malén Massot-Cladera, Ignasi Azagra-Boronat, Àngels Franch, Margarida Castell, Maria J. Rodríguez-Lagunas, Francisco J. Pérez-Cano

**Affiliations:** 1 Physiology Section, Department of Biochemistry and Physiology, Faculty of Pharmacy and Food Science, University of Barcelona (UB), 08028 Barcelona, Spain; malen.massot@ub.edu (M.M.-C.); ignasiazagra@ub.edu (I.A.-B.); angelsfranch@ub.edu (À.F.); margaridacastell@ub.edu (M.C.); mjrodriguez@ub.edu (M.J.R.-L.); 2 Nutrition and Food Safety Research Institute (INSA-UB), 08921 Santa Coloma de Gramenet, Spain

**Keywords:** inulin fiber, acacia fiber, immune system, microbiota, mineral absorption, IgA

## Abstract

The study’s objective was to ascertain whether a nutritional multivitamin and mineral supplement enriched with two different dietary fibers influences microbiota composition, mineral absorption, and some immune and metabolic biomarkers in adult rats. Nine-week-old Wistar rats were randomly assigned into four groups: the reference group; the group receiving a daily supplement based on a food matrix with proteins, vitamins, and minerals; and two other groups receiving this supplement enriched with inulin (V + I) or acacia (V + A) fiber for four weeks. Microbiota composition was determined in cecal content and mineral content in fecal, blood, and femur samples. Intestinal IgA concentration, hematological, and biochemical variables were evaluated. Both V + I and V + A supplementations increased *Firmicutes* and *Actinobacteria* phyla, which were associated with a higher presence of *Lactobacillus* and *Bifidobacterium* spp. V + A supplementation increased calcium, magnesium, phosphorus, and zinc concentrations in femur. V + I supplementation increased the fecal IgA content and reduced plasma total cholesterol and uric acid concentration. Both fiber-enriched supplements tested herein seem to be beneficial to gut-health, although differently.

## 1. Introduction

The intake in an appropriate dose (20–35 g/day for healthy adults) of dietary fiber (DF) has long been linked to reduction of metabolic diseases incidence, including diabetes, cardiovascular disease and obesity, among others, due to its capacity to lower blood cholesterol and C Reactive Protein (CRP), to attenuate glucose absorption and to improve insulin response [1,2,3]. Moreover, when non-digestible fiber reaches the colon unaltered and is selectively metabolized by microbiota, it induces specific changes, both in the composition and/or functionality of one or a limited number of bacteria potentially associated with health and well-being [4,5,6]. Meeting all these criteria, non-digestible fiber is considered a prebiotic as defined by Gibson and Roberfroid [1,4,5,6].

Prebiotic consumption is also believed to improve the immune system in both humans and animals [7]. The most examined mechanism involved in this effect is the indirect modulation of the immune response by changing the microbiota composition [8,9] and, therefore, its crosstalk with the immune system. Moreover, the enrichment of beneficial bacteria induced by prebiotic intake can result in the modulated release of pro- and anti-inflammatory cytokines [7] as well as in increasing the intestinal and fecal immunoglobulin (Ig) A content [10]. In addition, although little information is available, direct effects of prebiotics on the immune system such as changes of the intestinal gene expression, such as toll-like receptors (TLRs), have also been reported [11,12].

The favorable shift in the gut microbiota composition after prebiotic fiber consumption [1] is proposed as a potential mechanism by which prebiotics improve mineral absorption [13,14]. In this regard, the most widely accepted theory supporting this effect is associated with the microbial fermentation of the prebiotic fiber into short-chain fatty acids (SCFAs) in the colon. This metabolic process acidifies the intestinal compartment; thereby, preventing the formation of complexes between minerals and negatively charged metabolites. Consequently, it increases the extent of mineral absorption [15]. Alternatively, prebiotic consumption may also influence tissue morphology by increasing cell density, intestinal crypt depth, and blood flow in the large intestine, a mechanism that is believed to increase the intestinal surface area and lead to a higher mineral absorption [16,17].

There are several well-documented prebiotic fibers, such as the inulin-type fructans [18,19]. Inulin is part of everyday human diet. It can be found naturally among others in a range of plants such as chicory, garlic, tomato, and banana [20]. Its bifidogenic effects have been widely described in vitro, in vivo, and in clinical studies [19,21]. In the last few years, a new prebiotic fiber has emerged: acacia gum. It is a soluble DF obtained from the stems and branches of *Acacia senegal* and *Acacia seyal* and it is mainly composed of complex polysaccharides [22]. It resists digestion in the upper gastrointestinal tract; thus, reaching the large intestine and it can induce an increase in *Bifidobacterium* spp. in vitro [23,24] and in human [25] studies. However, unlike the inulin, little is known about the impact of acacia gum on health benefits. 

On the other hand, the maintaining of a healthy diet, defined as an appropriate balance of energy, macro- and micronutrients and water, is important for adults but it is particularly relevant for members of the elderly population who are more vulnerable to malnutrition. Moreover, the efficiency of nutrient absorption may be impaired in this population; thus, involving different nutritional requirements. Moreover, the presence of oral problems, for example with dentition, together with a decrease in smell and taste perception induce a change in dietary patterns. Additionally, there is a concomitant decline in the normal function of the immune system (immunosenescence) that may contribute to an increase in the risk of infection and frailty [26]. On this basis, the hypothesis of the present study is that the intake of a nutritional supplement containing proteins, vitamins, minerals, and fiber is beneficial for the adult population and if so, its impact should be important to take into account for the elderly. Therefore, the aim of the current study was to ascertain whether a nutritional multivitamin and mineral supplement enriched with two different DFs influences microbiota composition, mineral absorption, and some immune and metabolic biomarkers in adult rats. 

## 2. Materials and Methods 

### 2.1. Animals and Supplements

Nine-week-old female and male Wistar rats, purchased from Janvier Labs (Saint Berthevin Cedex, France), were housed individually in polycarbonate cages with large fibrous-particle bedding and tissue papers as enrichment, in a controlled environment of temperature and humidity and in a 12/12 h light/dark cycle at the Faculty of Pharmacy and Food Science animal facility. All rats were fed a commercial diet corresponding to the American Institute of Nutrition 93 M formulation [27] (Teklad Global 14% Protein Rodent Maintenance Diet, Envigo, Indianapolis, IN, USA), which contains 5% of cellulose, and water ad libitum throughout the study.

After the acclimation period, animals were randomly assigned into four experimental groups (*n* = 10/each, 5 females and 5 males). One constituted the reference (REF) group which did not receive any supplement; another group received a daily supplement based on a food matrix with proteins, vitamins and minerals (V) (Table S1); and two other groups received this supplement enriched with inulin (V + I) or acacia fiber (V + A), containing 4.5 g of fiber/100 g of product each (La Piara S.A, Manlleu, Barcelona). The chow consumption was measured every other day to adjust the dose of the supplements that were administered during 4 weeks in daily small portions. With this aim, the weights of each serving in V + I and V + A were readjusted periodically for each animal in order to receive an extra 20% of fiber daily. Accordingly, the same amount of supplement was used for the V group. 

All experimental procedures were approved by the Ethical Committee for Animal Experimentation of the University of Barcelona and the Government of Catalonia (CEEA UB ref. 351/17 and CG 9735, respectively), in accordance with the EU Directive 2010/63/EU.

With regard to sample size estimation (*n* = 10/group), the Appraising Project Office’s program from the Universidad Miguel Hernández de Elche (Alicante, Spain) was used to calculate the minimum number of animals providing statistically significant differences among groups, assuming that there is no dropout rate, a beta risk of 0.2 (80% power) and a type I error of 0.05 (two-sided). We used the IgA-coating bacteria percentage data from a previous study [11] with similar design: mean values in the REF group were 25.3%, the estimated common standard deviation was 13 and the minimum expected difference was 12. In addition, the sample size was adjusted to the minimum needed to follow the University Ethical Committee guidelines.

### 2.2. Monitoring, Sample Collection and Processing

Body weight and food and water intake were monitored three times per week throughout the study. Fecal samples were collected weekly in order to determine changes in the fecal wet weight, humidity, and pH. Additionally, fecal samples collected at the end of the study allowed the concentration of IgA and the proportion of IgA-coated bacteria to be quantified, as previously described [28], as well as the mineral elimination.

At the end of the nutritional intervention, animals were anesthetized intramuscularly with ketamine (90 mg/kg) (Merial Laboratorios, S.A. Barcelona, Spain) and xylazine (10 mg/kg) (Bayer A. G., Leverkusen, Germany) in order to obtain tissue samples. The body weight and naso–anal length were measured to calculate the body mass index (BMI) as body weight/length^2^ (g/cm^2^). Urine samples for mineral quantification were obtained by direct puncture of the bladder. The weight of stomach, duodenum, jejunum, ileum, cecum, colon and rectum, spleen, liver, thymus, kidneys, heart, submandibular gland, and the length of the small and large intestines were recorded. Blood samples were collected in heparin-treated tubes to determine the hematological and biochemical variables, and plasma mineral content. For IgA quantification, the gut wash (GW) from the distal part of the small intestine was obtained as previously described [11]. Finally, the central part of the left femur was excised for mineral quantification.

### 2.3. Fecal Variables

Fresh feces collected weekly were used to determine fecal pH using a surface electrode (Crison Instruments, S.A., Barcelona, Spain). Afterwards, fecal samples were dried for 24 h at 60 °C. Fecal humidity of each sample was calculated considering the weight difference between before and after the drying process.

### 2.4. Mineral Analysis in Biological Samples

Firstly, blood, feces, femur, and urine samples underwent a chemical cleavage process to obtain aqueous solutions without precipitation or colloids. For that, the same amount of each type of sample was introduced into a tared high-pressure vessel made of polytetrafluoroethylene. Then, 2 mL of concentrated nitric acid (HNO_3_) and 2 mL of concentrated hydrogen peroxide (H_2_O_2_) were also added in order to ensure a better oxidation of the organic matrix. All the high-pressure vessels were incubated overnight at 90 °C. After digestion, the samples were diluted with 16 mL of ultra-pure water, and then the vessels were weighed again in order to determine the weight of the aqueous sample solutions. Additionally, for each digestion cycle, triplicates of digestion blanks containing only HNO_3_, H_2_O_2_ and ultra-pure water were also prepared. Finally, the digested solutions were transferred into the test tubes. The concentrations of calcium (Ca), magnesium (Mg), phosphorous (P), and zinc (Zn) were determined using an inductively coupled plasma-optical emission spectrometer (ICP-OES, Optima 3200 RL, Perkin-Elmer, Massachusetts, USA), whereas iron (Fe) and zinc (Zn) concentrations were measured by an inductively coupled plasma-mass spectrometer (ICP-MS, Nexlon 350 D, Perkin-Elmer, Massachusetts, MA, USA) using standard conditions. The analysis was carried out at the Unit of Metal Analysis of the Scientific and Technological Centers of the University of Barcelona (CCiT-UB). Results are expressed as mg/g of sample.

### 2.5. Hematological and Biochemical Analysis

Heparin-treated blood was immediately used to count platelets and white and red blood cells and related variables using an automated hematology analyzer (Spincell3, MonLab, Barcelona), following the manufacturer’s instructions. 

Plasma samples were used to quantify total cholesterol (TC) by cholesterol oxidase (CHOD)-peroxidase (POD) method; high-density lipoprotein cholesterol (HDL-C) by colorimetric method; triglycerides (TG) by glycerol phosphate oxidase (GPO) method; glucose by glucose oxidase (GOD)-POD method and uric acid by Uricasa-POD method using kits provided by Química Clínica Aplicada, S.A. (Química Clínica Aplicada, S.A., Tarragona, Spain) and following the manufacturer’s instructions. Low-density lipoprotein cholesterol (LDL-C) was assessed according to the formula by Friedewald et al. in 1972, in which cLDL = TC – (cHDL + TG/5).

### 2.6. Immunoglobulin A and IgA-Coated Bacteria Quantification

The concentration of IgA in GW, fecal homogenates, and plasma was quantified at the end of the nutritional intervention by ELISA as previously described [11,29]. Moreover, the proportion of bacteria coated with IgA in feces was determined and analyzed by flow cytometry, as previously established [28]. The results concerning IgA are expressed as ng/g of tissue, ng/mg of fecal sample, and ng/mL of plasma, whereas those related to the IgA-coated bacteria are expressed as percentage.

### 2.7. Analysis of Cecal Microbiota Composition by 16S rRNA Sequencing

Cecal samples from all the animals at the end of the study (*n* = 10 animals/group) ranging from 500–1000 mg collected were used to extract genomic DNA using QIAmp DNA Stool Mini Kit (Qiagen) with some previous modification as has been previously reported [30]. Briefly, extra purification and concentration were performed following the cleaning protocol from QIAmp Micro Kit (Qiagen, Madrid, Spain). Then, for massive sequencing, the hypervariable region V3-V4 of the bacterial 16s rRNA gene was amplified using key-tagged eubacterial primers (forward: S-D-Bact-0341-b-S-17, 5′-CCTACGGGNGGCWGCAG-3′ and reverse S-D-Bact-0785-a-A-21, 5′-GACTACHVGGGTATCTAATCC-3′) [31] and sequenced with a MiSeq Illumina Platform (Illumina Inc., San Diego, CA, USA) by the Life Sequencing facilities (ADM Life Sequencing, Valencia, Spain), following the Illumina recommendation for Library preparation and sequencing for metagenomics studies. 

The software Paired-End read merger (PEAR v 0.9.6, Exelixis Lab, Heidelberg, Germany) was used to merge raw sequences forward and reverse. Using this approach, the ends of the obtained sequences were overlapped in order to get complete sequences. The amplification primers from the sequences obtained in the sequencing step were trimmed with Cutadapt v1.8.1 [32], using parameters by default, in order to reduce the bias in the annotation step. Once the primers were removed, sequences lower than 200 nucleotides were excluded from the analysis because short sequences have a higher chance of generating wrong taxonomical group associations. After obtaining the clean complete sequences, a quality filter was applied in order to delete sequences of poor quality. The resulting sequences were inspected for PCR chimera constructs (Uchime, USEARCH) [33], that may occur during the different experimental process, which were removed from further analysis. Later, each group of sequences was compared to a database of rRNA using an alignment BLAST strategy to associate taxonomic groups. The relative proportions of phyla, families, and genera were calculated. Moreover, to estimate the specific genus biodiversity, the Shannon–Wiener and CHAO1 indexes were calculated. 

Results of the qualitative analyses relative to the most abundant phyla, families, and genera are represented with stacked bars separated by gender. The category “others” represented in each graph includes those phyla whose presence was lower than 0.05% in the REF group; and those families and genera whose presence was lower than 0.8% in the same group.

To estimate the presence or absence of certain bacterial genera in the experimental groups, it was agreed that all bacterial genera present in all animals belonging to the same group with a proportion higher than 0.01% were computed as “present”. Otherwise, they were computed as “absent” in such groups. Based on that, the Venn diagrams were created for all groups together allowing the way the genera were distributed among the groups to be seen numerically, in order to compare their coincidences and differences.

### 2.8. Principal Components Analysis

The principal components analysis (PCA) was performed to evaluate the dimensionality of microbiota with regard to the supplements. The model was done using Simca v14.1 (Umetrics, Umeå, Sweden) as previously reported [30]. 

### 2.9. Statistical Analysis

The Statistical Package for the Social Sciences (SPSS v22.0) (IBM, Chicago, IL, USA) and the R software (R-3.6.3) were used for statistical analysis. Data were tested for homogeneity of variance and normality distribution by the Levene’s and Shapiro–Wilk tests, respectively. When data were homogeneous and normally distributed, a two-way ANOVA test was applied. When no differences between genders were observed, the data were analyzed together using a conventional-one-way ANOVA. Otherwise, they were analyzed separately. When significant differences among groups were detected, Bonferroni’s post hoc test was performed. Kruskal−Wallis test was used when results were neither equally nor normally distributed, followed by Nemenyi post hoc test in the case of significant difference among groups. To compare variables along the study, a repeated-measures ANOVA or Friedman test were applied followed by Student’s t-test or Nemenyi post hoc test, respectively. Significant differences were considered when *p* < 0.05, except regarding repeated comparisons, when *p* value was corrected, dividing it by the number of applied tests.

## 3. Results

### 3.1. Effects of Supplements on Morphometry and Food and Water Intake

Throughout the study period, male rats from all groups had a higher body weight (447.51 ± 5.80 g), chow intake (28.09 ± 0.68 g/day/rat), and water consumption (29.23 ± 0.56 mL/day/rat) than female rats (255.25 ± 2.5 g/day/rat, 17.73 ± 0.33 g/day/rat, and 21.66 ± 0.61 mL/day/rat, respectively) (*p* < 0.05), and none of the dietary supplementations modified these variables (Figure S1a,b). 

Regarding the body mass index (BMI) at the end of the nutritional intervention, only sex-associated significant differences were observed within all experimental groups. In particular, BMI in female rats was 0.64 ± 0.01 whereas it was 0.85 ± 2.5 in male rats, considering all animals independently of the experimental group, and no effects due to supplementation were detected (Figure S1c). 

Moreover, whereas male rats showed significantly lower relative weight in most of the organs analyzed than female rats in the same group (*p* < 0.05) (Table S2), supplementation did not result in changes. No differences between groups were found when the small intestine and large intestine were measured, their mean length being 80.69 ± 1.43 cm and 16.76 ± 0.34 cm, respectively, for all experimental groups considered together at the end of the study.

### 3.2. Effects of Supplements on Fecal Variables

No sex-associated differences were detected in all the fecal variables studied; thus, these results were analyzed considering both female and male rats together (Figure S2). 

Fecal weight registered was similar throughout the study and this was around 0.25 ± 0.01 g/day for all experimental groups, without being influenced by the dietary supplementations (Figure S2a). Similar results were observed when humidity of the feces was measured. All samples had around 53.24 ± 0.48% of water independently of the experimental group (Figure S2b). 

When pH was measured in fecal samples, it was similar during the first two weeks of supplementation, but higher pH was observed at the end of the study. This was not associated with any of the nutritional interventions given that the pH was similar (5.94 ± 0.04) in all experimental groups (Figure S2c).

### 3.3. Effects of Supplements on Mineral Concentration

Mineral content measured in blood, feces, femur, and urine samples at the end of the nutritional intervention for all experimental groups is summarized in Table 1.

In blood samples the most abundant mineral studied was iron, followed by phosphorus, calcium, and magnesium, whereas zinc was the one detected in the lowest concentration. Regarding the nutritional intervention, V + I and V + A-supplemented animals had lower calcium concentration compared to the group receiving non-fiber-enriched supplement (V group) (*p* < 0.05). Moreover, the supplement containing acacia fiber resulted in a lower magnesium blood concentration compared to the REF and V groups (*p* < 0.05).

The most abundant mineral detected in feces was calcium, followed by potassium, magnesium, and iron. No effects due to dietary intervention were identified in this compartment. 

When mineral content in femur was analyzed, the most abundant was calcium, followed by phosphorus, magnesium, and zinc. Iron was detected in a very low proportion. Interestingly, only acacia-enriched supplement significantly increased the concentration of calcium, magnesium, phosphorous, and zinc in comparison with both the V and V + I groups (*p* < 0.05). 

In urine samples the mineral found in most abundant concentration was magnesium, followed by calcium and potassium. Both zinc and iron were detected in very low concentrations. No effects due to dietary intervention were observed in this compartment.

### 3.4. Effects of Supplements on Hematological and Biochemical Variables

From all the parameters related to the leucocytes, erythrocytes, and platelets, only punctual differences in the erythrocyte parameters were seen (Table S3). The inulin-enriched supplement-fed animals (V + I) showed a slightly lower mean corpuscular hemoglobin (MCH) and a reduction in its concentration (MCHC) when compared to that of the REF and V groups (*p* < 0.05).

**Figure 1:**
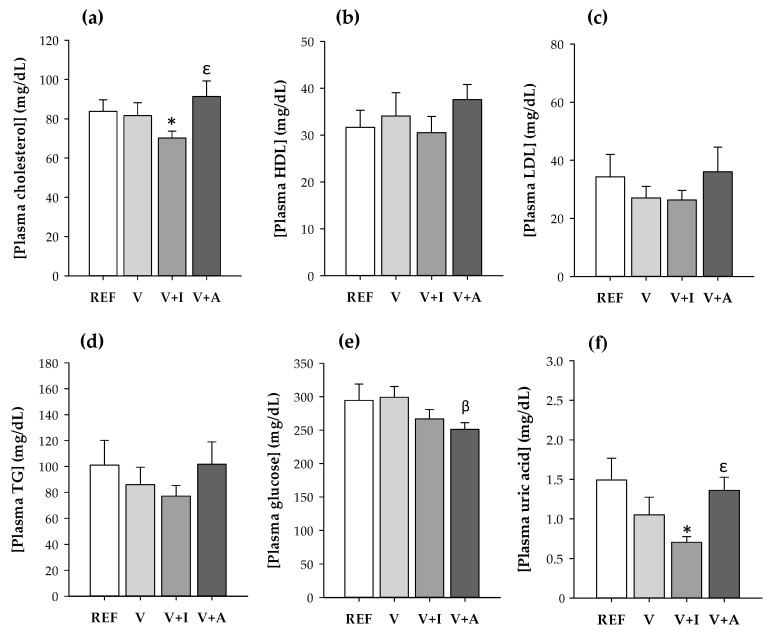
(**a**) Total cholesterol; (**b**) high-density lipoprotein cholesterol (HDL-C); (**c**) low-density lipoprotein cholesterol (LDL-C); (**d**) triglycerides (TG); (**e**) glucose; and (**f**) uric acid concentration in blood samples at the end of the study for all experimental groups considering both sexes together. Results are expressed as mean ± SEM (*n* = 10/group). Statistical significance: * *p <* 0.05 vs. REF group; ^β^ *p <* 0.05 vs. V group; ^ε^ *p <* 0.05 vs. V + I group.

The inulin-enriched supplement (V + I) intake resulted in a significantly lower plasma concentration of total cholesterol and uric acid in comparison to that of the REF group and to the acacia-supplemented animals (*p* < 0.05) (Figure 1a,f). Moreover, the acacia-enriched supplement intake reduced the glucose concentration compared to the supplement without fiber (*p* < 0.05) (Figure 1e).

No effects either on HDL-C, LDL-C, or triglycerides were found due to supplementation with the DF.

### 3.5. Effects of Supplements on IgA Concentration

**Figure 2:**
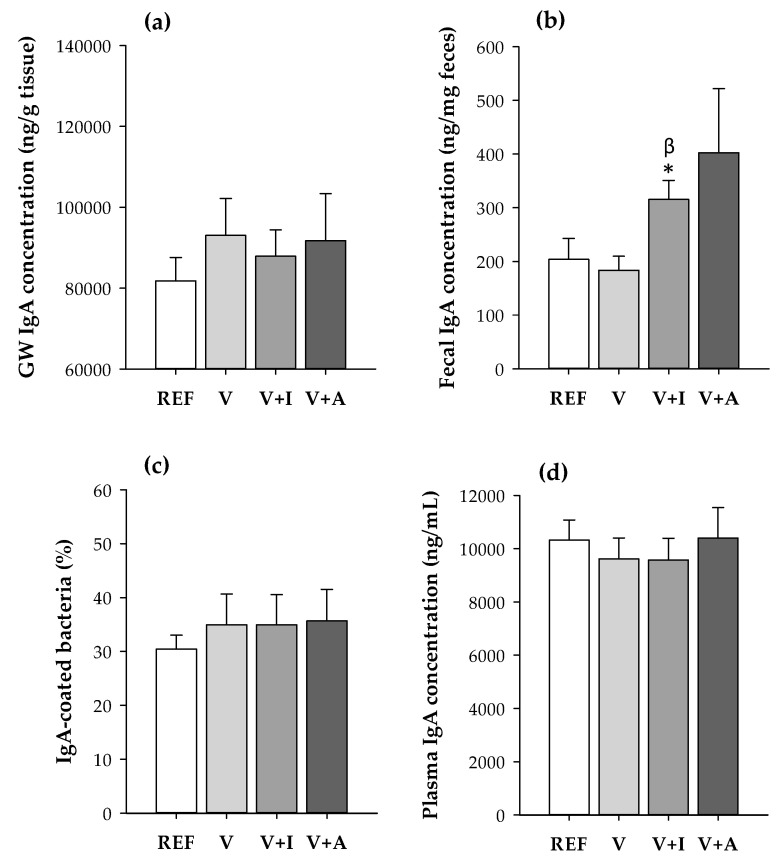
(**a**) IgA concentration in gut wash (GW) and (**b**) fecal samples, (**c**) proportion of fecal IgA-coating bacteria and (**d**) plasma IgA concentration quantified at the end of the study for all experimental groups considering both sexes together. Results are expressed as mean ± SEM (*n* = 10/group). Statistical significance: * *p <* 0.05 vs. REF group; ^β^ *p <* 0.05 vs. V group.

The mean fecal IgA content was 1.5–2-fold higher in animals receiving both the inulin- (V + I) and acacia-enriched (V + A) supplements, compared to the REF and V groups (Figure 2). However, only the increase caused by inulin was statistically significant compared to both the REF and V groups (*p* < 0.05).

No changes due to dietary intervention were detected either in the gut wash and plasma IgA concentrations or in the proportion of fecal IgA-coated bacteria. 

### 3.6. Effects of Supplements on Cecal Microbiota Composition

#### 3.6.1. Diversity and Taxonomic Analysis

No changes on the Shannon–Wiener Index (3.52 ± 0.06) and CHAO1 (448.13 ± 10.69), as indicators of the diversity and richness of the microbial community, respectively, were observed after any dietary supplementation for all experimental groups at the end of the study. 

**Figure 3:**
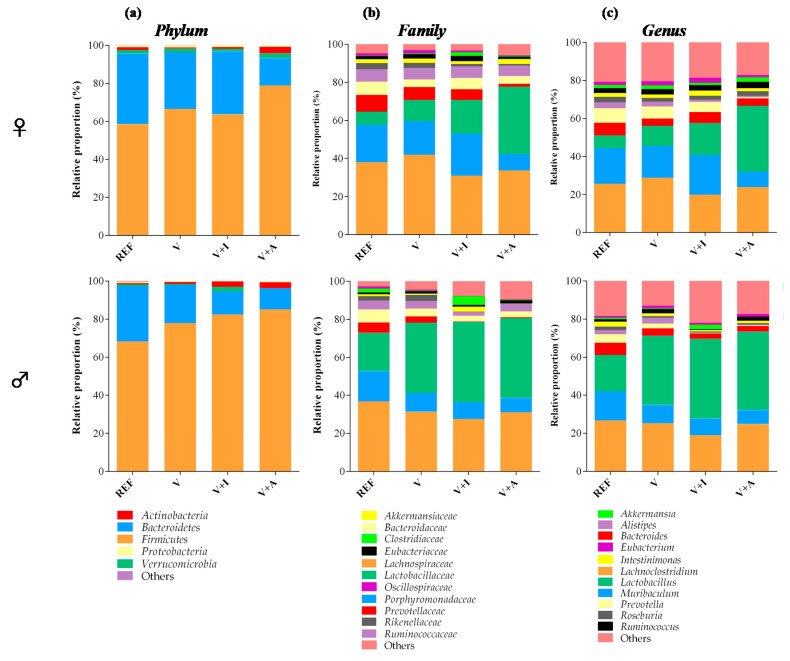
Main taxonomic ranks showing the proportion of bacterial populations in the cecal content at the end of the study in males and females. The relative proportion of the bacteria was calculated in each taxonomic rank: (**a**) phylum, (**b**) family, and (**c**) genus. Results are expressed as mean (*n* = 5 female or male/group). Significant differences not shown.

Although it is well established that *Firmicutes* and *Bacteroidetes* are the most abundant phyla in the cecal microbiota, male rats had a lower proportion of *Bacteroidetes* in favor of that of Firmicutes. This increase in *Firmicutes* in males was also associated with a higher proportion of the family *Lactobacillaceae* spp. and in particular, the genus *Lactobacillus* (Figure 3).

**Figure 4:**
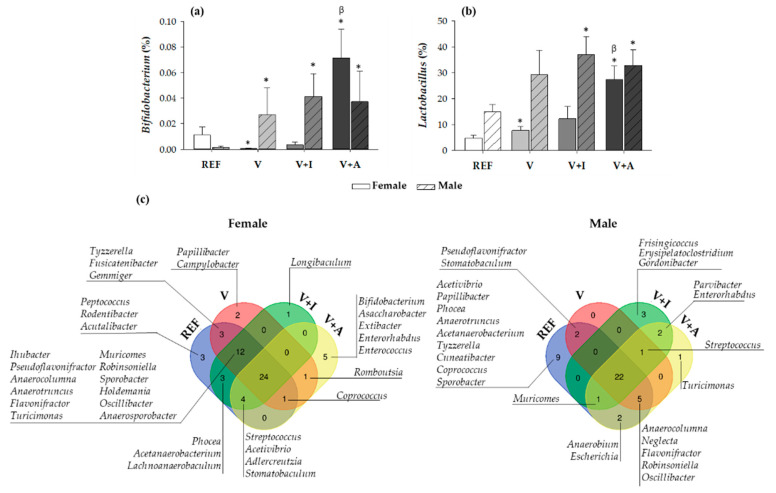
The relative proportion of the (**a**) *Bifidobacterium* and (**b**) *Lactobacillus* genera in cecal content at the end of the study differentiating between sexes. Results are expressed as mean ± SEM (*n* = 10/group). Statistical significance: * *p* < 0.05 vs. REF group; ^β^ *p* < 0.05 vs. V group. (**c**) A representation of Venn diagrams showing the diversity in all genera differentiating between sexes. Results derived from *n* = 10/group.

Regarding the nutritional intervention, the acacia-enriched supplement (V + A) increased the proportion of the *Firmicutes* (up to 81.76%) and decreased *Bacteroidetes* (up to 12.92%), compared to the REF group whose proportions were 63.28% and 33.29%, respectively (*p* < 0.05) (Figure 3a). These changes were more evident in female than in male rats. These changes after acacia fiber-enriched supplement (V + A) intake were associated with an increase of *Lactobacillaceae* family, this effect being stronger in female than in male rats (Figure 3b), whose *Lactobacillaceae* proportion was already increased at the baseline. Moreover, acacia supplementation significantly increased genera belonging to the *Firmicutes* and *Actinobacteria* phyla (Figure 3c). In particular, a significant increase in *Bifidobacterium* spp. (up to 0.07% and 0.06% in females and males, respectively) and *Lactobacillus* spp. (up to 28% and 33% in females and males, respectively) was observed in V + A-fed animals in comparison with those in the REF (< 0.015% and < 15% for *Bifidobacterium* spp. and *Lactobacillus* spp., respectively, in both females and males) and V (< 0.03% and < 30% for *Bifidobacterium* spp. and *Lactobacillus* spp., respectively, in both females and males) groups (*p* < 0.05) (Figure 4a,b). 

#### 3.6.2. Venn Diagrams and Principal Components Analysis: Genera

The analysis of the genera distribution in Venn diagrams revealed that there was a core of 24 and 22 genera, in female and male rats, respectively, that persisted in all four experimental groups when considering both sexes separately (Figure 4c). Moreover, when comparing the microbiota depending on the supplementation, it could be observed that both the inulin (V + I) and acacia-enriched supplements (V + A) were able to exclusively promote the colonization of new genera in both female and male rats. On the one hand, inulin was able to promote the colonization of one new genus (*Longibaculum*) in female rats and five new genera in male rats (*Frisingicoccus, Erysipelatoclostridium, Gordonibacter, Parvibacter,* and *Enterorhabdus*), two of which also appeared with acacia supplementation. On the other hand, acacia supplementation resulted in the colonization of five new genera (*Bifidobacterium, Asaccharobacter, Extibacter, Enterorhabdus,* and *Enterococcus)* in female rats and three new genera in male rats (*Streptococcus*, *Parvibacter*, and *Enterorhabdus*), two of which also appeared in the inulin. In addition, some particular genera present in both the REF and V groups were absent in the V + I and V + A groups, this being the case in eight for females and eleven for males.

**Figure 5:**
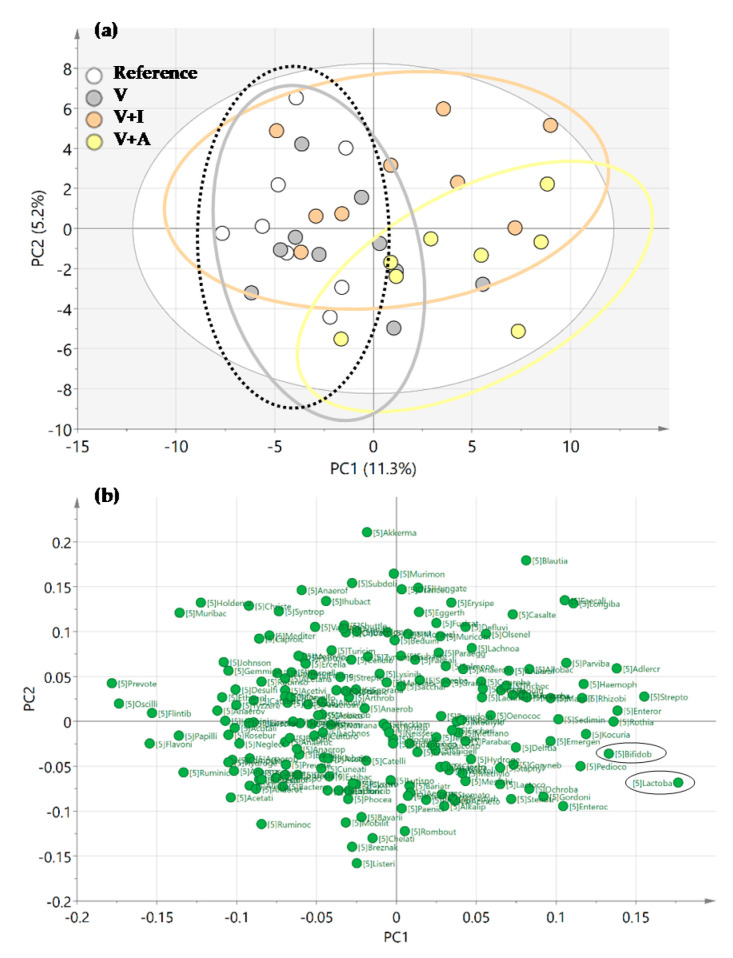
(**a**) Representation of Principal Components Analysis (PCA) for all experimental groups in a score plot and (**b**) a loading plot. Results derived from *n* = 10/group.

The PCA score plot revealed that the microbiota of the acacia-fiber supplemented animals (V + A) clustered differentially compared to those of both the REF and V groups in the genera analysis (Figure 5a). Moreover, the loading plot revealed that the *Bifidobacterium* spp. and *Lactobacillus* spp. were variables involved in the clustering of the V + A group (Figure 5b). 

## 4. Discussion

Changes in dietary pattern, implying a lower intake of vitamins and minerals, may lead also to changes in microbiota composition. This situation, besides having a poor nutrient absorption and reduced bacterial diversity, is even more evident in the elderly [34]. In the current study, adult rats have been used to mimic the feasible impact of the fiber-enriched nutritional supplements tested herein on the immunological, hematological, and biochemical variables. Moreover, due to the disparity existing in the immune response, microbiota composition and the susceptibility to disease between gender [35,36,37], both female and male rats have been included in the present interventional study. 

Herein, we demonstrate that the supplementations with inulin, a well-known prebiotic, and acacia gum fibers modify the adult rat microbiota composition with different intensity. Rats aged nine weeks that received acacia gum supplementation daily for four weeks showed an increased proportion in *Firmicutes* and *Actinobacteria.* In this regard, the acacia supplementation resulted in a significantly higher presence of *Lactobacillus* and the appearance of *Bifidobacterium* in both genders. In fact, this is not the first time that acacia gum’s potential as a prebiotic agent has been described in both in vitro [38] and clinical studies [22,25]. Indeed, an interventional study carried out in human volunteers demonstrated that consumption of 10 and 15 g/day of acacia gum for 10 days increased the counts of both lactic acid-producing bacteria and *Bifidobacterium* in feces [22]. Moreover, its ability to selectively prevent the overgrowth of unwanted bacteria such as *Clostridium difficile* or *Clostridium histolyticum,* has also been studied in vitro [38,39], although some controversy exists. 

Moreover, acacia fiber supplementation produced the appearance of the genus *Asaccharobacter* (which belongs to the *Actinobacteria* phylum) in female rats, a single species of which has been reported to be a powerful equol producer [40]. Therefore, older people receiving the acacia fiber-enriched supplement may benefit from equol’s health-promoting benefits, as has been reported, for example on osteoporosis, prostate cancer, and cardiovascular diseases [41,42]. 

In this study, the appearance of the genus *Enterorhabdus* (*Actinobacteria* phylum) has been associated with the consumption of acacia supplementation in both female and male rats and also with the inulin supplement in male rats. Although little is known about the possible role of this *Actinobacteria* genus, its higher relative abundance has been negatively correlated to serum TC, TG and LDL-C and hepatic TC, TG, bile acids, and non-esterified fatty acids in *Grifola frondosa* polysaccharide-chromium III-treated type 2 diabetes mellitus (T2DM) mice [43]. Although further, more in-depth studies should be carried out into this association, it seems that *Enterorhabdus* genus could exert some kind of hypoglycemic and hypolipidemic activities in T2DM, one of the multifactorial chronic metabolic disorders affecting mainly adults worldwide. Therefore, this result suggests that the inclusion of acacia or inulin fiber in the diet of adult people would be beneficial for them.

One of the objectives of the present study was to compare the prebiotic activity of both inulin and acacia fibers in adult rats. Surprisingly, the effects observed on microbiota composition after acacia fiber intake in female rats were significantly stronger than those exerted by inulin in the same gender. These differential results agree with those reported by Calame et al. who evidenced that acacia gum was able to produce a higher increase in both bifidobacteria and lactobacilli than an equal dose of inulin in healthy men [25].

The shift in microbiota composition has been proposed as a potential mechanism by which prebiotics improve mineral absorption [13,14]. In the current study, increased calcium, magnesium, phosphorus, and zinc concentrations were observed in femur from both female and male acacia-supplemented animals; thus, suggesting that acacia-enriched supplement could be beneficial for bone mineralization. Similar findings to those described herein have been reported after galactooligosaccharides (GOS), fructooligosaccharides (FOS), a mixture of GOS/FOS, and inulin supplementation in vitro, in vivo, and in human studies [15,44]. However, the fact that in our study no effects were observed in the inulin-supplemented group could be due to an insufficient dose, the type of inulin used, or the duration of the treatment. On the other hand, either the promotion of the lactic-acid bacteria or the production of SCFA may result in an acidification of the colon compartment; thus, preventing the formation of complexes between mineral and negatively charged metabolites, and therefore improving the bioavailability of minerals [15]. In the present study we found no changes in either fecal or cecal pH after the dietary supplementations that would explain this mechanism. The lack of intestinal acidification after inulin intake has already been reported in younger rats fed a diet with inulin for three weeks [28]. Further studies should be carried out in order to elucidate the impact of both fiber supplementations on SCFA production and their relationship with the mineral absorption in rats.

Most research has only been focused on the indirect effects of prebiotics over a considerable period of time. However, it has recently been evidenced that prebiotics may also cause direct effects, such as immunomodulation in the gastrointestinal tract [45]. In this regard, it is well known that prebiotic administration, such as inulin, generally results in increased fecal IgA concentration [18]. This fact is in line with those results obtained here because the intake of inulin-enriched supplement for four weeks increased fecal IgA content; thus, enhancing the intestinal immune system, with no difference between genders. With regard to the acacia-enriched supplement-fed animals, although a relevant tendency to increase fecal IgA levels was observed, it did not reach statistical significance. None of the dietary supplementations tested herein modified the proportion of IgA-coated bacteria, contrary to what was observed in the youngest rats whose proportion increased after three weeks of inulin intake [28]. Further studies should confirm these results and should be aimed at understanding them. 

On the other hand, fiber and prebiotic intake in appropriate doses is associated with less incidence of metabolic diseases due to its indirect capacity to modulate the blood lipid profile and other metabolic variables [46]. In the current study, the intake of the inulin-enriched supplement for four weeks exerted lipid-lowering effects by significantly reducing the cholesterol and the uric acid in plasma. These results are partially in line with those reported in animal models [47] and in hypercholesterolemic [48] and healthy [49,50] subjects receiving an inulin supplementation for 3–16 weeks. Nevertheless, the modulation of biochemical variables after acacia fiber intake is quite controversial. Whereas some authors did not observe significant effects either in hypercholesterolemic [51] or in healthy [52] subjects, others have attributed to acacia fiber significant benefits for metabolic disorders [53,54]. In particular, the intake of 30 g of acacia for three months resulted in a significant reduction of blood triglyceride and fasting plasma glucose concentrations in type 2 diabetic patients [53]. However, the conditions (dose and length) tested within the study evidenced a tendency to reduce the glucose and uric acid concentrations. This lack of effect after the acacia-enriched supplement on biochemical variables agrees with that reported in hypercholesterolemic [51] or in healthy [52] subjects. Further studies are required to clarify the protective effects of acacia gum on cardiometabolic diseases.

## 5. Conclusions

Overall, both fiber-enriched supplements tested in the present study show the potential to be beneficial to gut-health, although differently. Whereas inulin-enriched supplement shows intestinal immune enhancement, acacia fiber supplement has stronger prebiotic activity, which may lead to increasing mineral absorption.

## Figures and Tables

**Table 1:** Mineral concentration (mg/g) in blood, feces, femur, and urine samples at the end of the study for all experimental groups considering both sexes together.

		[Ca]	[Fe]	[Mg]	[P]	[Zn]
		(mg/g)	(mg/g)	(mg/g)	(mg/g)	(mg/g)
Blood	**REF**	60.11 ± 2.51	0.45 ± 0.01	34.36 ± 1.10	0.37 ± 0.01	0.005 ± 0.000
	**V**	63.45 ± 2.20	0.44 ± 0.01	32.28 ± 0.76	0.35 ± 0.01	0.005 ± 0.000
	**V + I**	57.21 ± 3.16 ^β^	0.44 ± 0.00	31.80 ± 0.68	0.34 ± 0.01	0.005 ± 0.000
	**V + A**	59.53 ± 4.24 ^β^	0.44 ± 0.01	31.73 ± 0.36 ^*β^	0.34 ± 0.01	0.005 ± 0.000
Feces	**REF**	19.35 ± 1.43	0.41 ± 0.03	3.70 ± 0.14	12.39 ± 0.61	0.19 ± 0.01
	**V**	18.55 ± 1.21	0.39 ± 0.02	3.67 ± 0.15	12.02 ± 0.51	0.19 ± 0.01
	**V + I**	18.16 ± 1.36	0.43 ± 0.04	3.57 ± 0.04	12.13 ± 0.59	0.19 ± 0.01
	**V + A**	19.96 ± 1.04	0.39 ± 0.02	3.87 ± 0.13	13.20 ± 0.50	0.21 ± 0.01
Femur	**REF**	140.60 ± 6.33	0.05 ± 0.00	3.00 ± 0.13	66.95 ± 2.93	0.15 ± 0.01
	**V**	126.03 ± 8.05	0.06 ± 0.01	2.71 ± 0.15	61.61 ± 3.40	0.14 ± 0.00
	**V + I**	120.05 ± 6.73	0.05 ± 0.00	2.60 ± 0.16	58.93 ± 2.90	0.14 ± 0.00
	**V + A**	157.90 ± 4.77 ^βε^	0.05 ± 0.00	3.45 ± 0.10 ^βε^	76.87 ± 2.33 ^βε^	0.16 ± 0.00 ^βε^
Urine	**REF**	0.08 ± 0.03	0.002 ± 0.001	0.33 ± 0.12	0.04 ± 0.02	0.001 ± 0.000
	**V**	0.11 ± 0.04	0.001 ± 0.000	0.30 ± 0.08	0.13 ± 0.11	0.003 ± 0.003
	**V + I**	0.08 ± 0.02	0.000 ± 0.000	0.23 ± 0.05	0.04 ± 0.02	0.002 ± 0.001
	**V + A**	0.17 ± 0.07	0.001 ± 0.000	0.33 ± 0.10	0.02 ± 0.00	0.001 ± 0.001

Results are expressed as mean ± SEM (*n* = 10/group). Calcium and magnesium blood concentrations are expressed as the mean ± SEM of mg of each mineral × 10^−3^/g of sample. REEF: animals no receiving supplement; V: animals receiving a daily supplement based on a food matrix with proteins, vitamins and minerals; V + I: inulin-enriched supplement-fed animals, and V + A: acacia-enriched supplement-fed animals.* *p <* 0.05 vs. REF group; ^β^ *p <* 0.05 vs. V group; ^ε^ *p <* 0.05 vs. V + I group. Ca: calcium; Fe: iron; Mg: magnesium; P: phosphorus; Zn: zinc.

## Supplementary Materials

The following are available online at https://www.mdpi.com/2072-6643/12/8/2196/s1, Figure S1: (**a**) Body weight and (**b**) food intake registered throughout the study and (**c**) body mass index (BMI) measured at the end of the study differentiating between sexes. Results are expressed as mean ± SEM (*n* = 5/group). No significant differences were observed. Figure S2: (**a**) Fecal wet weight, (**b**) humidity, and (**c**) pH registered throughout the study considering both sexes together. Results are expressed as mean ± SEM (*n* = 10/group). No significant differences were observed. Table S1: Composition and content of macronutrients and micronutrients (fiber, vitamins, and minerals) of the experimental supplements. Table S2: Relative weight of organs expressed as percentage (%) with respect to the body weight at the end of the study for all experimental groups differentiating between sexes. Results are expressed as mean ± SEM (*n* = 5 female and male/group). Statistical significance: ^α^ *p <* 0.05 vs. REF. Table S3: Hematological parameters in blood samples at the end of the study for all experimental groups considering both sexes together. Results are expressed as mean ± SEM (*n* = 10/group). Statistical significance: * *p <* 0.05 vs. REF group; ^β^ *p <* 0.05 vs. V group; ^ε^ *p <* 0.05 vs. V + I group.
