# Supplementary material for: Gut Health-Promoting Benefits of a Dietary Supplement of Vitamins with Inulin and Acacia Fibers in Rats
Source: Nutrients. 2020 Jul 23;12(8):2196. doi: 10.3390/nu12082196 (PMC7468733; doi:10.3390/nu12082196)
Supplement: Supplementary file 1 [file nutrients-12-02196-s001.pdf]

Figure S1

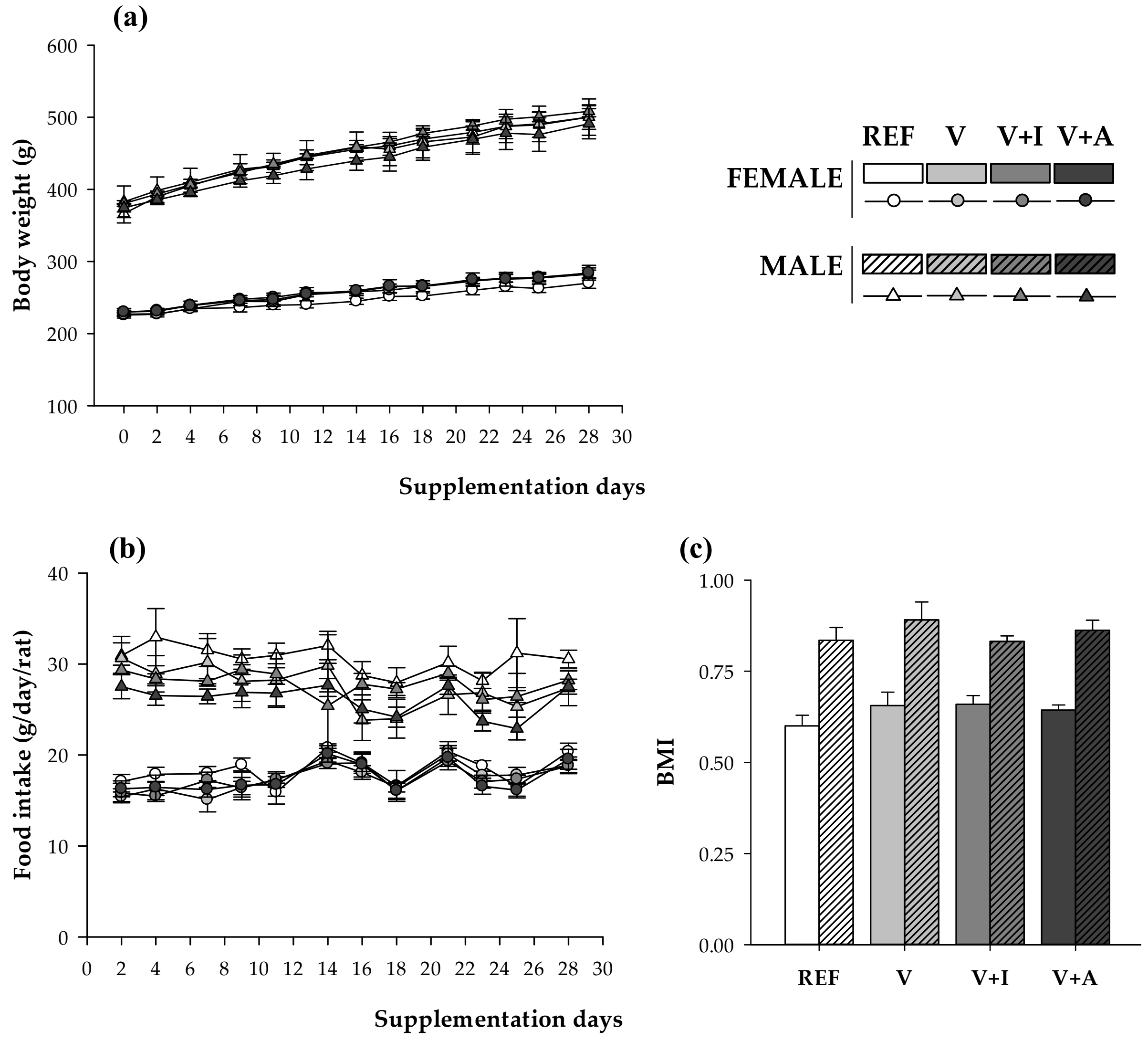
(a)
Body weight (g)
600
500
400
300
200
100
0
2
4
6
8
10
12
14
16
18
20
22
24
26
28
30
Supplementation days
REF
V
V+I
V+A
FEMALE
MALE
(b)
Food intake (g/day/rat)
40
30
20
10
0
Supplementation days
(c)
BMI
1.00
0.75
0.50
0.25
0.00
REF
V
V+I
V+A

Figure S2

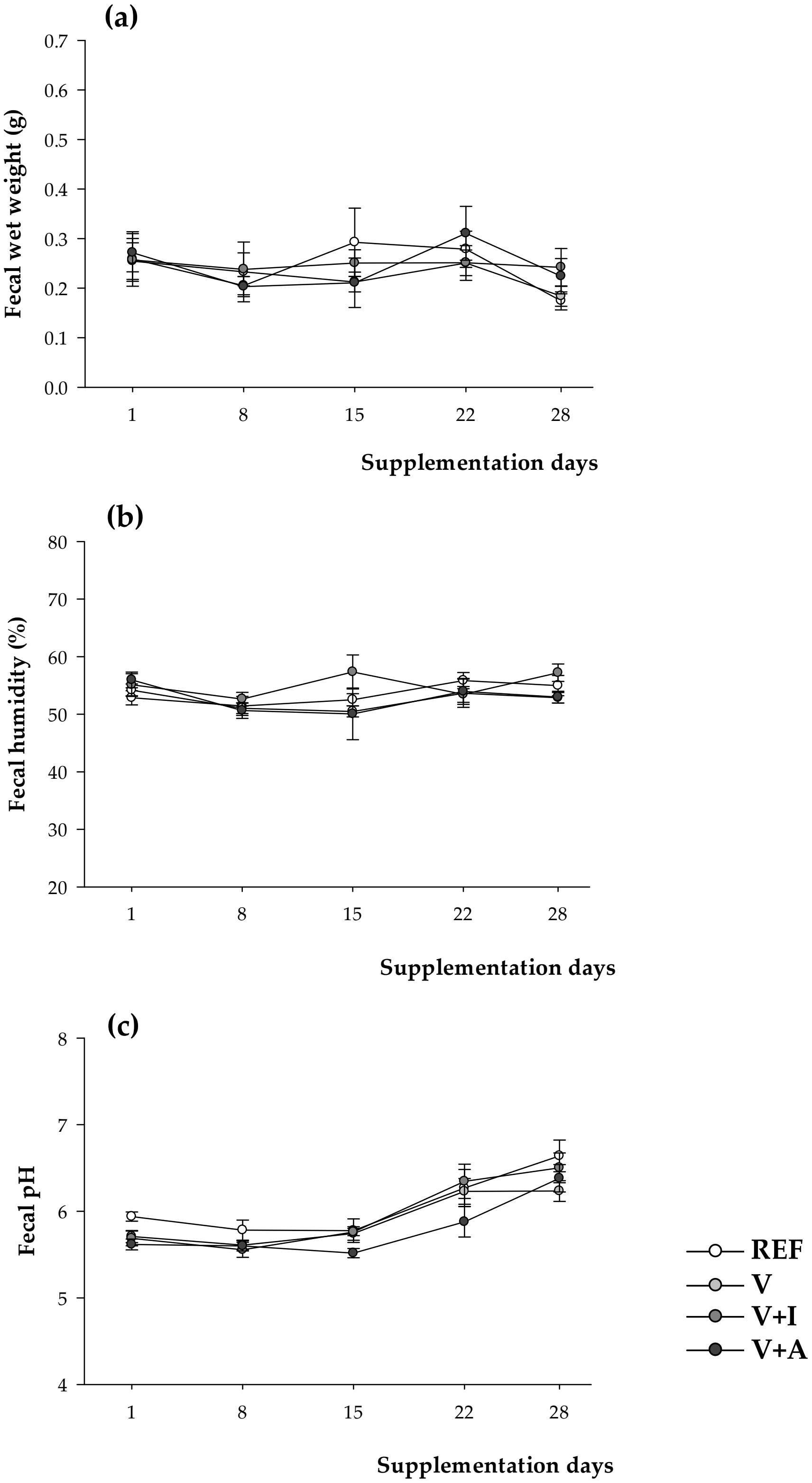
(a)
Fecal wet weight (g)
0.0
0.1
0.2
0.3
0.4
0.5
0.6
0.7
1
8
15
22
28
Supplementation days
(b)
Fecal humidity (%)
20
30
40
50
60
70
80
1
8
15
22
28
Supplementation days
(c)
Fecal pH
4
5
6
7
8
1
8
15
22
28
Supplementation days
REF
V
V+I
V+A

Table S1

**Components**	**V**	**V+I**	**V+A**
Kcal/100 g	110.82	114.10	110.90
Proteins (%)	9.45	8.90	8.80
Carbohydrate (%)	6.73	7.00	7.00
Sugar (%)	1.3	3.40	3.30
Fat (%)	4.9	4.50	4.50
Saturated (%)	0.94	0.86	0.87
Fiber (g/100 g)	1.0	4.50	4.50
Soluble (g)	0.15	4.50	3.40
Sodium (mg/100 g)	374,5	410.00	411.00
Calcium (mg/100 g)	140.00	140.00	170.00
Iron (mg/100 g)	2.30	2.40	3.10
Selenium (mg/100 g)	0.005	0.005	0.005
Zinc (mg/100 g)	2.60	2.70	2.20
Vitamin B_6(mg/100 g)	0.38	0.31	0.35
Vitamin B_9 (mg/100 g)	0.03	0.03	0.03
Vitamin B_{12} (mg/100 g)	<0.0004	<0.0004	<0.0003
Vitamin D (mg/100 g)	0.0004	0.0005	0.0007
Vitamin E (mg/100 g)	3.39	3.26	3.37

Table S2

RELATIVE WEIGHT (%)	REF		V		V+I		V+A	
	♀	♂	♀	♂	♀	♂	♀	♂
**Stomach**	0.52 ± 0.03	0.40 ± 0.02$^{\alpha}$	0.54 ± 0.04	0.44 ± 0.02	0.53 ± 0.01	0.41 ± 0.02$^{\alpha}$	0.49 ± 0.03	0.40 ± 0.03$^{\alpha}$
**Duodenum**	0.16 ± 0.01	0.10 ± 0.01$^{\alpha}$	0.16 ± 0.01	0.11 ± 0.01$^{\alpha}$	0.17 ± 0.02	0.11 ± 0.01$^{\alpha}$	0.16 ± 0.01	0.11 ± 0.01$^{\alpha}$
**Jejunum**	1.37 ± 0.02	0.97 ± 0.06$^{\alpha}$	1.24 ± 0.02	0.97 ± 0.04$^{\alpha}$	1.20 ± 0.09	0.94 ± 0.02$^{\alpha}$	1.25 ± 0.05	0.97 ± 0.03$^{\alpha}$
**Ileum**	1.05 ± 0.07	0.77 ± 0.02$^{\alpha}$	1.10 ± 0.05	0.83 ± 0.05$^{\alpha}$	1.09 ± 0.02	0.84 ± 0.03$^{\alpha}$	0.96 ± 0.06	0.76 ± 0.05$^{\alpha}$
**Cecum**	0.36 ± 0.04	0.32 ± 0.06	0.31 ± 0.03	0.31 ± 0.05	0.33 ± 0.04	0.33 ± 0.06	0.32 ± 0.04	0.28 ± 0.02
**Colon & rectum**	0.52 ± 0.04	0.53 ± 0.03	0.53 ± 0.05	0.41 ± 0.02	0.53 ± 0.02	0.38 ± 0.02$^{\alpha}$	0.48 ± 0.02	0.35 ± 0.03$^{\alpha}$
**Spleen**	0.29 ± 0.02	0.23 ± 0.01$^{\alpha}$	0.31 ± 0.02	0.22 ± 0.01$^{\alpha}$	0.30 ± 0.02	0.24 ± 0.01$^{\alpha}$	0.28 ± 0.03	0.19 ± 0.02$^{\alpha}$
**Liver**	3.67 ± 0.10	3.72 ± 0.09	3.59 ± 0.18	3.90 ± 0.19	3.39 ± 0.12	3.76 ± 0.13	3.66 ± 0.07	3.78 ± 0.09
**Thymus**	0.22 ± 0.02	0.15 ± 0.01$^{\alpha}$	0.24 ± 0.01	0.13 ± 0.01$^{\alpha}$	0.23 ± 0.03	0.15 ± 0.01$^{\alpha}$	0.23 ± 0.01	0.15 ± 0.02$^{\alpha}$
**Kidneys**	0.68 ± 0.01	0.55 ± 0.02$^{\alpha}$	0.66 ± 0.04	0.60 ± 0.03	0.63 ± 0.02	0.58 ± 0.02	0.62 ± 0.02	0.59 ± 0.02
**Heart**	0.39 ± 0.01	0.31 ± 0.02$^{\alpha}$	0.39 ± 0.01	0.31 ± 0.01$^{\alpha}$	0.39 ± 0.01	0.31 ± 0.01$^{\alpha}$	0.36 ± 0.01	0.31 ± 0.01$^{\alpha}$
**Submandibular gland**	0.10 ± 0.02	0.07 ± 0.00	0.08 ± 0.00	0.01 ± 0.00$^{\alpha}$	0.08 ± 0.00	0.07 ± 0.00$^{\alpha}$	0.08 ± 0.01	0.07 ± 0.00

Table S3

		REF	V	V+I	V+A
WBC[a]	**GB (x10^9/L)**	5.81 ± 0.75	5.33 ± 0.62	4.94 ± 0.56	5.98 ± 0.79
	**LYM (%)**	57.40 ± 1.85	60.82 ± 1.73	55.94 ± 2.04	56.74 ± 2.08
	**MID (%)**	7.08 ± 0.22	7.26 ± 0.16	7.45 ± 0.20	7.38 ± 0.21
	**GRAN (%)**	35.52 ± 1.79	31.84 ± 1.64	36.26 ± 2.20	34.88 ± 2.68
	**LYM# (x10^9/L)**	3.37 ± 0.53	3.20 ± 0.38	2.67 ± 0.31	3.41 ± 0.55
	**MID# (x10^9/L)**	0.37 ± 0.06	0.34 ± 0.05	0.29 ± 0.04	0.40 ± 0.06
	**GRAN#(x10^9/L)**	2.07 ± 0.20	1.81 ± 0.22	1.93 ± 0.26	2.17 ± 0.22
RBC[b]	**GR (x10^12/L)**	7.22 ± 0.15	7.03 ± 0.14	7.37 ± 0.10	7.24 ± 0.18
	**HGB (g/dL)**	12.72 ± 0.26	12.44 ± 0.20	12.48 ± 0.13	12.44 ± 0.10
	**HCT (%)**	39.10 ± 0.87	37.69 ± 0.99	40.06 ± 0.51	38.93 ± 0.79
	**MCV (fL)**	54.27 ± 0.94	54.06 ± 0.69	54.45 ± 0.81	54.25 ± 1.02
	**MCH (pg)**	17.58 ± 0.26	17.82 ± 0.21	16.38 ± 0.49$^{*\beta}$	17.30 ± 0.44
	**MCHC (g/dL)**	32.50 ± 0.28	33.13 ± 0.54	31.12 ± 0.47$^{*\beta}$	31.99 ± 0.62
	**RDW-CV (%)**	11.30 ± 0.20	11.40 ± 0.16	10.30 ± 1.02	11.65 ± 0.15
	**RDW-SD (fL)**	20.37 ± 0.33	20.28 ± 0.23	20.61 ± 0.14	20.74 ± 0.34
Platelet	**PLQ (x10^9/L)**	365.44 ± 34.23	351.83 ±16.08	351.80 ± 17.51	331.40 ± 42.12
	**MPV (fL)**	9.47 ± 0.38	9.15 ± 0.22	9.64 ± 0.27	10.08 ± 0.70
	**PDW (fL)**	10.53 ± 0.57	9.62 ± 0.34	11.01 ± 0.59	10.58 ± 0.61
	**PCT (%)**	0.33 ± 0.03	0.32 ± 0.01	0.33 ± 0.02	0.30 ± 0.03
	**P-LCR (%)**	11.07 ± 1.31	9.75 ± 0.94	11.64 ± 0.97	11.57 ± 1.27
	**P-LCC (x10^9/L)**	37.67 ± 3.87	34.28 ± 2.77	40.80 ± 4.19	32.62 ± 3.83

[a]WBC= White Blood Cells; [b]RBC= Red Blood Cells.

White blood cell count (**GB**); Lymphocyte percentage **(LYM**); Monocyte percentage (**MID**); Granulocyte percentage (**GRA**); Lymphocyte count (**LYM#**); Monocyte count (**MID#**); Granulocyte count (**GRA#**); Red blood cell count (**GR**); Hemoglobin concentration (**HGB**); Hematocrit (**HCT**); Mean Corpuscular Volume (**MCV**); Mean Corpuscular Hemoglobin (**MCH**); Mean Corpuscular Hemoglobin Concentration (**MCHC**); Red Blood Cell Distribution width Repeat Precision (**RDW-CV**); Red Blood Cell Distribution Width STDEV (**RDW-SD**); Platelet count (**PLT#**), Mean platelet volum (**MPV**); Platelet distribution width (**PDW**); Plateletcrit (**PCT**); Large platelet ratio (**P-LCR**); Large platelet (**P_LCC**).
